# The Role of Radiation Therapy in Unicentric Castleman Disease: A Case Report

**DOI:** 10.7759/cureus.49687

**Published:** 2023-11-29

**Authors:** Cristina Rodriguez, Lesbia Rivera Rubi, Oscar Menjivar, Juan Suazo

**Affiliations:** 1 Clinical Oncology, Toluca State Oncology Center, Toluca, MEX; 2 Clinical Oncology, Pedregal's Angels Hospital, Mexico City, MEX; 3 Oncology, Yucatan's Higher Speciality Hospital, Merida, MEX; 4 Oncology, Leeds Teaching Hospitals, Leeds, GBR

**Keywords:** hyaline vascular, pelvic mass, multidisciplinary treatments, r-chop-rituximab, multicentric castleman disease, unicentric castleman’s disease

## Abstract

Castleman disease (CD) is a rare lymphoproliferative disorder characterized by localized (unicentric) or systemic (multicentric) lymphadenopathy. This study presents a unique case of a 29-year-old female with a rare pelvic presentation of unicentric Castleman disease, specifically the hyaline vascular variant. Despite surgical resection, an unresectable residual lesion prompted adjuvant radiotherapy and subsequent chemotherapy. The literature highlights surgical resection as the primary treatment for localized Castleman disease; however, radiotherapy and combined chemotherapy regimens like cyclophosphamide, doxorubicin, vincristine, and prednisone (CHOP) have shown promise in unresectable cases, emphasizing a multidisciplinary approach. This case underscores the importance of tailoring treatment strategies for uncommon Castleman disease presentations.

## Introduction

Castleman disease (CD), also known as angiofollicular lymph node hyperplasia, portrays a diverse collection of lymphoproliferative disorders with shared histopathologic characteristics [1]. The disease was first described by Dr. Benjamin Castleman in 1956 when he reported cases of localized mediastinal lymph node hyperplasia resembling thymoma [2].

The categorization of CD is based on two variables: the number of regions of enlarged lymph nodes with distinctive histopathologic characteristics and concomitant human herpesvirus 8 (HHV-8) infection. Regarding the number of regions, CD can be divided into two distinct diseases: unicentric CD (UCD) and multicentric CD (MCD) [1].

Unicentric CD is defined as one or more enlarged lymph nodes in a particular area of the body. The histopathologic features of UCD range from the hyaline vascular subtype to a mixed in-between to the plasmacytic subtype. In addition, some UCD patients can present with systemic symptoms [1,3].

Patients with UCD often remain asymptomatic, with the disease typically identified during a physical examination or through imaging studies. After surgical removal of the affected lymph node, patients are monitored annually with laboratory tests, including a complete blood count (CBC), lactate dehydrogenase (LDH), chemistries with liver and renal function assessments, electrolytes, interleukin-6, albumin, serum free light chain assay, quantitative immunoglobulins, and C-reactive protein [1,4].

The diagnosis of UCD is suspected in patients presenting with a solitary, continuously enlarged lymph node that shows moderate to intense post-contrast enhancement on the computed tomography. Further evaluation with 18F-fluorodeoxyglucose (FDG) positron emission tomography (PET) is recommended. With regards to PET, it should be used to determine if the disease is restricted to a particular location with a relatively lower standardized uptake value (SUV) in other areas. If the SUV is more than 10, lymphoma should be considered a differential diagnosis instead of UCD [1].

In order to diagnose UCD, an excisional biopsy with a histopathologic review should show an enlarged lymph node. Also, detection of HHV-8 through immunohistochemistry for LANA-1 of lymph node tissue should be completed as part of the investigations for CD [1].

The multicentric CD is described as multiple areas of lymphadenopathy with histopathologic features similar to UCD. These patients also have systemic inflammatory symptoms and can have generalized lymphadenopathy, cytopenia, hepatosplenomegaly, and organ failure [1,5].

The disease's hallmark, progressive lymphadenopathy, seems to stem from an imbalance in IL-6 production. While its exact cause remains elusive, theories suggest roles for immunological processes [6]. Recent findings also associate HHV-8, EBV, and HIV with the disease's origin, suggesting potential viral factors [7]. Due to its association with HHV-8, Rituximab monotherapy has been shown to be effective, achieving a full response in cases of HHV-8-positive multicentric Castleman’s disease [1,5].

## Case presentation

**Figure 1:**
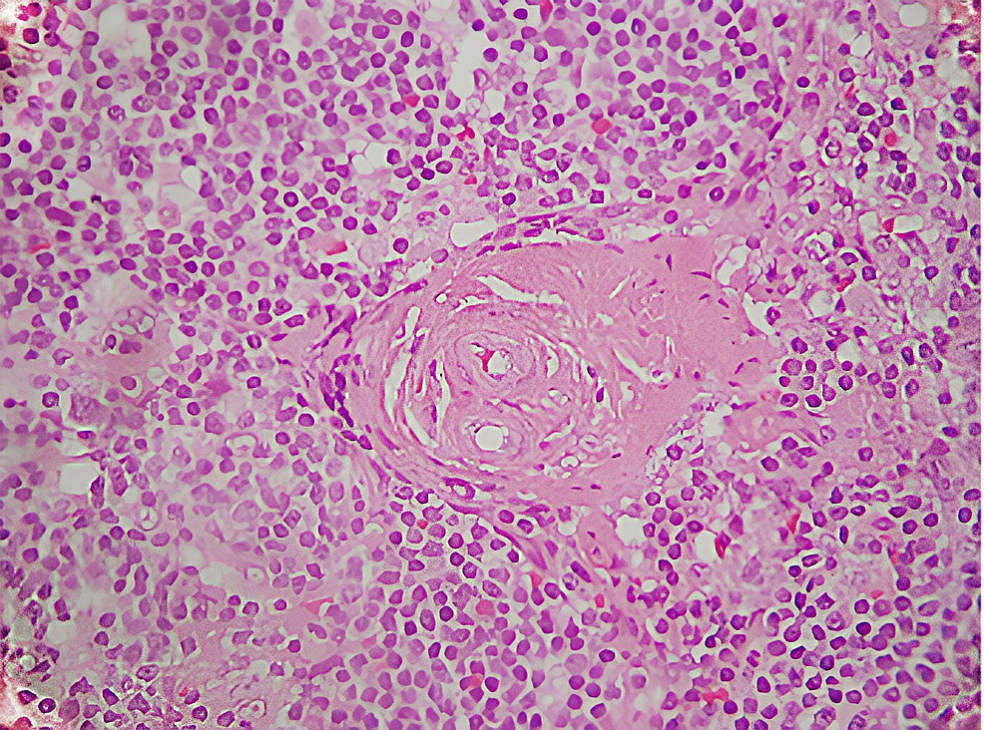
Microscopy shows HE staining displaying follicular hyperplasia.

A 29-year-old female, previously healthy, presented with pelvic pain of sacral predominance. Initial evaluation through abdominal ultrasound revealed a pre-sacral tumor. A subsequent CT abdomen-pelvis guided a transabdominal tumor resection, confirming Castleman disease, a hyaline vascular variant, with incomplete resection and a residual lesion size of 8 x 6.5 x 4 cm (Figure 1).

**Figure 2:**
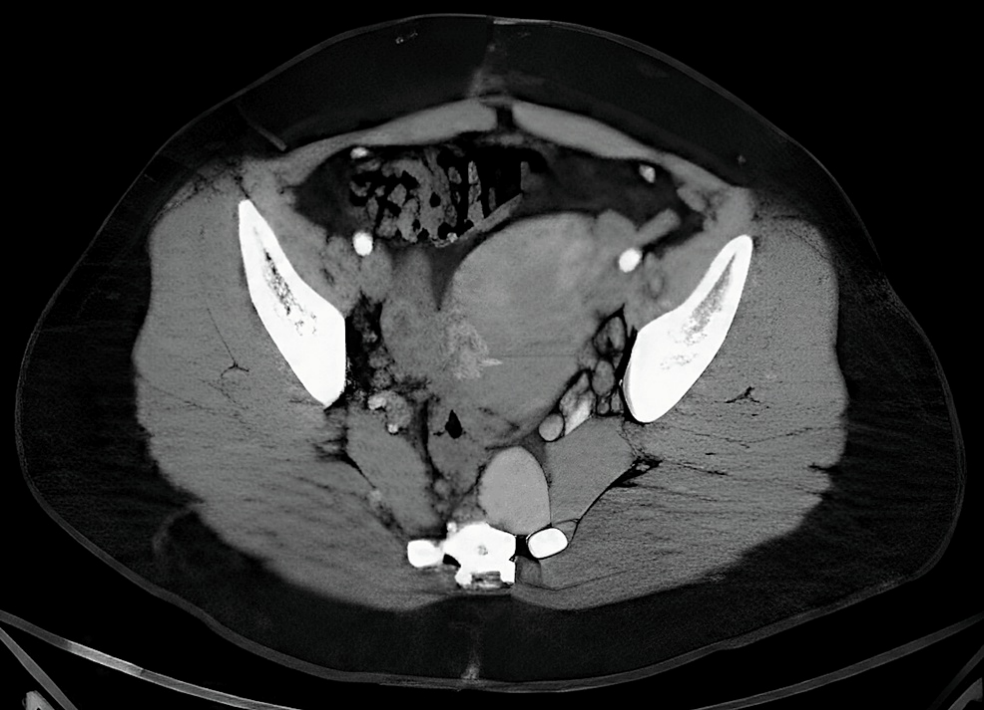
A CT scan of the abdomen and pelvis showed a homogeneous, solid left perirectal lesion.

Immunohistochemical analysis revealed a characteristic pattern (CD20+, CD5+, CD21+, CD34+). Further staging CT identified a left perirectal solid nodular lesion and nonspecific nodes in the left internal iliac chain (Figure 2).

Given the persistent and unresectable nature of the disease, the patient underwent 25 fractions of radiation therapy (50 Gy) using 18MV photons via a four-field technique. Unfortunately, post-treatment surveillance revealed continued disease presence, leading to subsequent cycles of CHOP-21 chemotherapy, with stabilization of the local disease noted in a follow-up CT a year later.

## Discussion

Among Castleman's diseases, Unicentric Castleman's disease is the most prevalent, occurring at a rate of 16 cases per million person-years, while Idiopathic Multicentric Castleman Disease is the least common, with an incidence of five cases per million person-years. UCD can present at any age, with a median age of presentation of 34 years and a wide age range from 2 to 84 years old, with female predominance [8]. Our patient is female and close to the mean age of 29 years old.

UCD presents with localized lymphadenopathy in an asymptomatic person, with common areas affected, including the mediastinum, axillary, inguinal regions, and abdominal cavity [6]. In this case, ultrasound and CT confirmed the presence of a pre-sacral tumor, perirectal lesion, and nodes in the left internal iliac chain. Similarly, in the case of the UCD hyaline vascular variant reported by Chen C. and Hsin-Ni Li, the initial investigations included an ultrasound followed by a CT and further MRI, which was not done in our patient. Immunohistochemical analysis in this case reported positive CD20 and CD21 with negative CD3 and Bcl-2 in contrast to our findings (CD20+, CD5+, CD21+, CD34+) [9].

For patients with unicentric CD, surgical resection is considered the standard therapy, often achieving high success rates with complete resolution of symptoms and laboratory changes. Recurrences are rare and are due to incomplete initial resection, missed lymph nodes, or a missed diagnosis of multicentric CD. In a systematic review of 278 published cases, disease-free survival rates at three and five years after surgery were 90 and 81 percent [7].

In 1972, Keller et al. reported that radiotherapy, administered at doses between 1800 and 4300 centigrays (cGy), yielded a minimal reduction in lymphadenopathy. Consequently, a consensus emerged within the medical community that radiotherapy lacked efficacy [10]. In 2000, a patient treated with radiotherapy alone reported a minimal decrease in tumor size and remained asymptomatic [11]. Currently, radiotherapy has emerged as a viable therapeutic option, with few cases reported.

Although many lesions remain stable and asymptomatic, radiotherapy (administered at doses of 30 to 45 Gy) can effectively treat lesions displaying regional disease progression or those deemed unresectable. The response rates can be as high as 40%, depending on the specific series [1]. Sethi et al. reported a full recovery in a patient with systemic symptoms who received 4000 cGy of treatment for the unicentric-HV variant [12].

Chronowski et al. presented four cases of UCD where patients were treated with two fractionation schedules of radiotherapy (200 cGy per day to a total dose of 4000 cGy and 180 cGy per day to a total dose of 3960 cGy) with a median follow-up of 30 months. Three of the four patients presented with complete radiographic resolution of the lymphadenopathy. Two of the patients are alive and clinically free of disease, and two died of unrelated events [4].

Additional research presents eight patient cases with unicentric disease treated using radiotherapy. Out of these cases, three patients experienced complete lymphadenopathy resolution, two became symptom-free but retained some lymphadenopathy, one patient's tumor size decreased, another had a limited response to therapy, and one patient saw disease recurrence after initially resolving. These patients received radiation doses ranging from 2700 to 4500 cGy. Notably, there was no clear-cut correlation between the radiation dose and either tumor response or symptom relief. However, it is worth mentioning that most patients who achieved a favorable outcome received approximately 4000 cGy of radiation [4].

In this case, CT transabdominal resection resulted in an incomplete resection with a nodular lesion and nonspecific nodes in the internal iliac chain. In situations like the present case, where complete surgical removal is not attainable or has only been partially accomplished, the addition of radiotherapy alongside surgery becomes a viable alternative. The patient received 25 fractions of radiation therapy (5000 cGy) using 18MV photons via a four-field technique, higher than the previously reported cases, but still presented persistent disease presence.

In addition to radiotherapy for cases where surgery is not an option, Rituximab, which is primarily used for multicentric CD, has shown promising results in patients with unresectable unicentric CD [1,5]. Modern four-drug combined chemotherapy regimens such as CHOP (cyclophosphamide, doxorubicin, vincristine, and prednisone) or CVAD have also exhibited sustained benefits, with over 50% of patients achieving a 10-year remission [4].

In a retrospective analysis of 33 patients who were not suitable candidates for surgery, 5 out of 8 individuals exhibited a partial response to chemotherapy, and 2 out of 5 showed a partial response to rituximab [1]. In this case, the patient underwent multiple cycles of CHOP-21 chemotherapy, and a follow-up CT scan conducted one year later revealed stability in the local disease.

## Conclusions

Surgical resection remains the primary treatment strategy for unicentric Castleman disease. However, radiotherapy and chemotherapy offer a reasonable alternative, achieving good response rates in patients not suitable for surgery or those with incomplete surgical resection. Further research and larger studies are needed to determine the optimal treatment approach for this rare disease.
